# Treating acute rhinitis and exacerbations of chronic rhinitis – A role for topical decongestants?

**DOI:** 10.4102/safp.v62i1.5053

**Published:** 2020-03-24

**Authors:** Robin J. Green, Charles Feldman, Andre van Niekerk, Marinda McDonald, Raymond Friedman, Guy A. Richards

**Affiliations:** 1 Department of Paediatrics and Child Health, University of Pretoria, Pretoria, South Africa; 2 Departments of Critical Care and Internal Medicine, University of the Witwatersrand, Johannesburg, South Africa; 3 Private Practice, Netcare Clinton Clinic, Alberton, Gauteng, South Africa; 4 The Allergy Clinic, Blairgowrie, Johannesburg, South Africa; 5 Private Practice, Mediclinic, Sandton, Johannesburg, South Africa

**Keywords:** acute rhinitis, nasal congestion, topical nasal decongestants

## Abstract

Acute nasal symptoms are troublesome for patients. In addition, these symptoms are encountered frequently by individuals because of common infectious diseases, especially rhinovirus, giving rise to a ‘common cold’. Acute nasal symptoms include rhinorrhoea, sneezing, nasal itch and congestion. Of these, nasal congestion is the most irritating. Because topical nasal decongestants provide rapid and dramatic relief from these symptoms, especially nasal congestion, they are frequently used and abused by patients. Guidance for indications, choice of most efficacious decongestant and recommendations for limiting side effects are thus essential to be imparted to patients by doctors.

## Introduction

Acute nasal symptoms are particularly troublesome for patients. These symptoms are encountered frequently by individuals because of common infectious diseases, not only those caused by rhinovirus but also because of other respiratory viruses, which give rise to a ‘common cold’. Acute nasal symptoms include rhinorrhoea, sneezing, nasal itch and congestion, and of these, nasal congestion is the most irritating manifestation for patients.

Because topical nasal decongestants provide rapid and dramatic relief from these symptoms, especially nasal congestion, they are frequently used and abused by patients. As such, it is essential that doctors provide guidance regarding their indications, choice of the most efficacious agents and recommendations for limited side effects.

### Terminology

**FIGURE 1:**
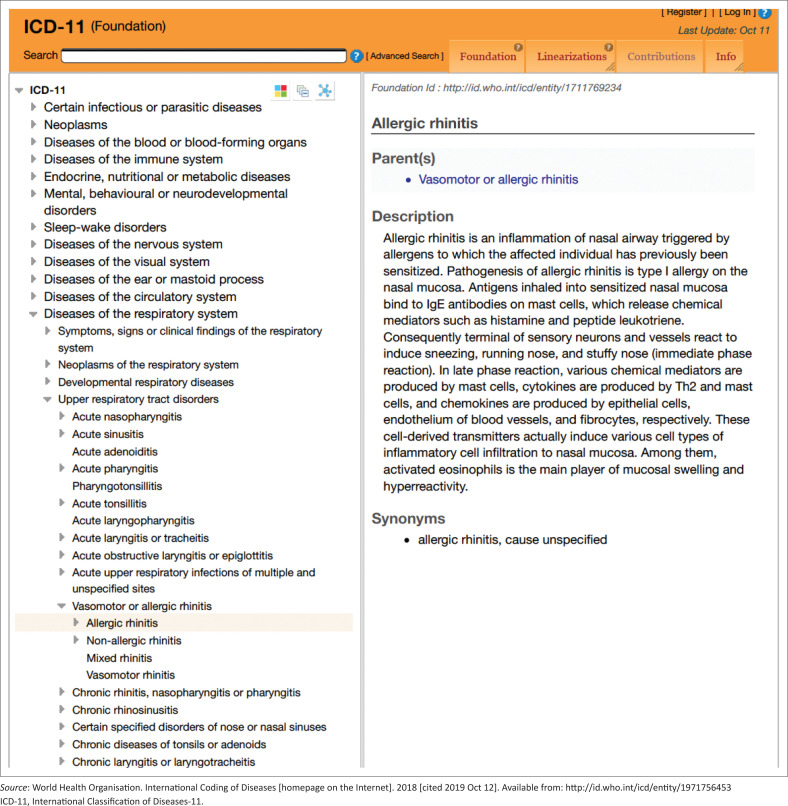
International Classification of Diseases-11.

It is critical that acute rhinitis is diagnosed correctly and precisely. The updated International Classification of Diseases (ICD)-11, which codes for and describes common conditions of the upper respiratory tract, is useful in this regard, providing clarity on definitions of diseases^1^ (Figure 1). For example, non-allergic rhinitis is described as ‘an inflammation of nasal mucosa in which allergic mechanisms are not involved’. It also covers different phenotypes listed as ‘non-allergen-induced rhinitis, rhinitis of the elderly, non-allergic non-infectious rhinitis’, etc.

The description of ‘cold’ is provided under the terminology ‘acute nasopharyngitis’ (Box 1).

BOX 1 International Classification of Diseases-11. Definition of acute nasopharyngitis.^1^

**Acute nasopharyngitis**

Upper respiratory tract disorders

Infections caused by rhinovirus

Other and unspecified diseases of the respiratory system

**Description**

A disease of the upper respiratory tract caused by infection with rhinovirus and other viruses. This disease is characterised by pharyngitis, running nose, stuffy nose or cough. Transmission is by inhalation of infected respiratory secretions or direct contact.

**Additional information**

Common cold (also known as nasopharyngitis, rhinopharyngitis, acute coryza or simply ‘cold’) is a viral infection of the upper respiratory track, which affects primarily the nose. The meaning of the nasopharynx is the same as the epipharynx; however, the term nasopharyngitis is generally used when epipharyngitis extends to the nose, pharynx and larynx. Ninety per cent of ‘colds’ are caused by viral infections, whilst the remaining 10% are caused by occasional bacterial or mycoplasma infections. Patients with nasopharyngitis present with cough, pharyngeal pain and running or stuffy nose as local symptoms, and increasing fever, general fatigue and headache as general symptoms. These symptoms usually resolve in 7–10 days, with some symptoms lasting for up to 3 weeks.

*Source:* World Health Organisation. International Coding of Diseases [homepage on the Internet]. 2018 [cited 2019 Oct 12]. Available from: http://id.who.int/icd/entity/1971756453

## Aetiology of acute rhinitis

**FIGURE 2:**
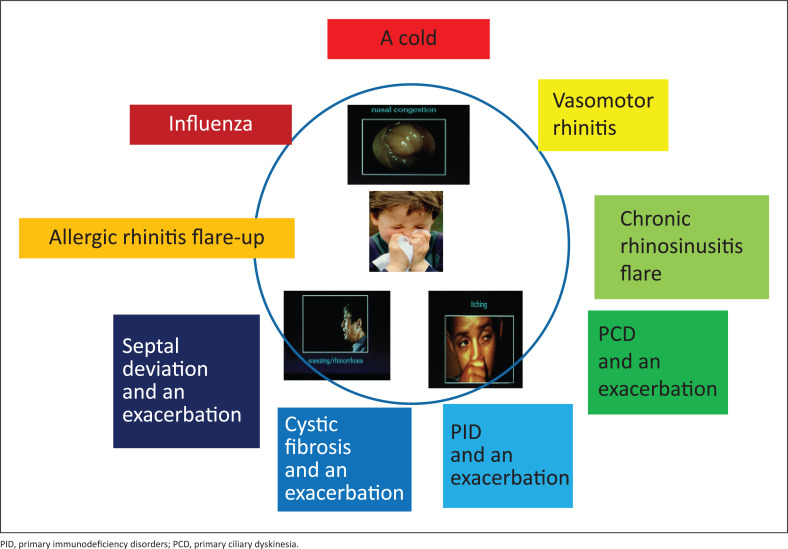
Aetiology of acute rhinitis symptoms.

Many causes of persistent nasal symptoms are well described and include allergic rhinitis (AR) and vasomotor rhinitis, cystic fibrosis, primary immunodeficiency disorders (PID), primary ciliary dyskinesia, chronic infectious rhinosinusitis and structural conditions. However, underlying chronic rhinitis frequently manifests with acute exacerbations of symptoms that mimic the common causes of an acute rhinitis such as acute nasopharyngitis (Figure 2). It is very important to distinguish an exacerbation of an underlying common condition from a benign viral ‘cold’.

### History and examination are important

Any child or adult who presents to a health care provider with acute rhinitis needs to be asked some important questions and must have an appropriate examination (Box 2). Examination must also distinguish whether symptoms are of a new event or represent an exacerbation of an underlying condition.

BOX 2 A thorough history and examination for acute rhinitis.

History includes:

Duration

Predominant symptoms

Associated pathologies (e.g. cough, wheezing, conjunctivitis)

Recurrent symptoms – how frequent?

Triggering phenomena

Sick household contacts

Sinister symptoms (e.g. epistaxis, mass, weight loss)

Examination must include:

The face

The nasal mucosa

The throat

The tympanic membranes

Sinus tenderness

The conjunctivae

The chest

**FIGURE 3:**
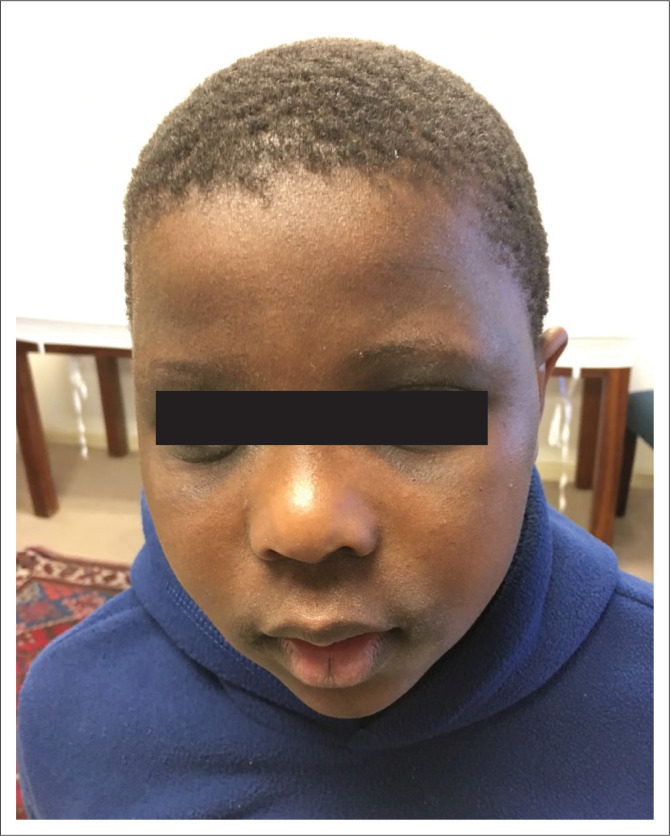
Features of an allergic face. (With permission from the child and parent.)

In the case of AR, the facial appearance of the patient is often the clue to the aetiology of acute symptoms (Figure 3). These features include dark rings beneath the eyes (allergic shiners), a nasal crease, double folds of the lower eyelids (Dennie–Morgan folds), open mouth breathing (teeth and gums show) and often allergic mannerisms (such as frequent nasal wiping). Allergic rhinitis may often present with quality of life impairment, including sleep deprivation and attention deficit disorder.^2^

### Treating nasal symptoms

If the nasal symptoms are caused by an underlying condition, then that condition needs to be addressed with both specific diagnosis and directed therapy. An important example is that if the patient has frequent nasal and other problems caused by a PID, then that condition needs a specific diagnosis and therapy if indicated.

As almost all acute rhinitides are troublesome, patients seek a number of over-the-counter remedies. Many of those are ineffective and have the potential to cause unwanted side effects or in fact prolong the duration of nasal congestion.

Some of the appropriate therapies for acute rhinitis, depending on aetiology, include oral and topical antihistamines, topical intranasal steroids and saline (and variants). However, the most effective agent to have rapid relief from symptoms is a topical nasal decongestant, and it is because of the immediate relief provided by this form of therapy that there is a high potential for overuse. The excessive use of topical decongestants may result in side-effects and failure to seek advice for a potential chronic underlying condition. Importantly, antibiotics (topical and systemic) are not indicated for managing acute rhinitis.^3^ Systemic decongestants, alone or in combination with other medications, have limited value for acute rhinitis and often have unacceptable side effects, especially in children.^4^ Systemic side effects include nervousness, insomnia, irritability, headache, palpitations and tachycardia. In addition, they may elevate blood pressure and intraocular pressure, and induce urinary obstruction.^5^

### Which topical nasal decongestant?

The most important criterion for selecting a nasal decongestant is evidence for efficacy and safety. The imidazole group of agents such as oxymetazoline and xylometazoline, which have a proven efficacy and safety profile, best fit these requirements (Box 3).^6,7,8,9,10,11,12,13,14,15,16^

BOX 3 The ideal topical nasal decongestant should have the following properties.

- A quick onset of action. In a prospective, double-blind, placebo-controlled clinical trial, oxymetazoline-metered nose spray (0.05 %) demonstrated a statistically significant 2-day reduction in the duration of acute rhinitis versus physiological saline, with a mean onset of action in 25 s.^7^
- A prolonged duration of action, preferably up to 12 h.^8^
- Belongs to the imidazoline group, regarded as the safest class of nasal decongestants.^6^
- Be safe and legal for use in athletes in competitive sports.^6^
- Be safe to be used for patients of all age groups.^9^
- Preferably have some *in vitro* anti-inflammatory properties.^10^
- A shown efficacy in vasomotor rhinitis.^11^
- An added decongestant effect when combined with nasal steroids.^12,13,14^
- Proof of reduction in nasal congestion.^15,16^

s, seconds; h, hours.

### Duration of use of topical decongestant

When used alone, topical nasal decongestants may produce rebound vasodilation in some patients after as little as 5-day use. As such, they should not, as a rule, be used for more than 7–10 days.^6,17^

One additional issue regarding safety of topical decongestants is related to the mode of delivery. Whereas metered dose sprays are safe, at least one study has documented that when using liquid solutions, an excessive volume may be delivered when the bottle is inverted, and therefore the dispenser must be kept upright.^18^

### Patient education

A critical step in managing rhinitis is education of the patient, the public and the provider, including the pharmacist, the primary health care practitioner and even the specialist.

It may also be worthwhile to provide a patient suffering from chronic rhinitis an ‘exacerbation plan’, which provides information as to the use of a topical nasal decongestant. It should also provide instructions as to the administration of the spray. Because most of the congestion occurs at the posterior and lateral side wall of the nose, it should be directed intranasally towards the ipsilateral ear.

The key principles of an education message are listed in Box 4.

BOX 4 Key educational messages in treating rhinitis.

- Blocked and runny noses have a cause.
- Topical decongestants treat the symptoms, not the cause.
- Topical decongestants are very effective in treating the symptoms, but should not be used for more than a week to 10 days.
- If nasal symptoms go on beyond a week, the topical decongestant should be stopped and the patient should consult a doctor who should identify and treat the cause.
- Do not make use of topical decongestants that are mixed into solutions with antibiotics and corticosteroids.
- Avoid oral decongestants either alone or in combination with other medication.

## Conclusion

Intranasal decongestants are effective in managing the troublesome symptoms of acute rhinitis, either because of a minor viral illness or an exacerbation of an underlying serious chronic condition. They do not however treat the cause, and if used over a prolonged period without further evaluation of the patient, a more important diagnosis could be missed out. Furthermore, they may cause rebound rhinitis medicamentosa.
